# Understanding the effect of HF-based wet shallow etching on optical performance of reactive-ion-etched fused silica optics†

**DOI:** 10.1039/d1ra04174f

**Published:** 2021-09-01

**Authors:** Laixi Sun, Ting Shao, Xinda Zhou, Weihua Li, Fenfei Li, Xin Ye, Jin Huang, Shufan Chen, Bo Li, Liming Yang, Wanguo Zheng

**Affiliations:** Research Center of Laser Fusion, China Academy of Engineering Physics Mianyang 621900 P. R. China yexin@caep.cn; School of Materials Science and Engineering, Southwest University of Science and Technology Mianyang 621010 P. R. China; IFSA Collaborative Innovation Center, Shanghai Jiao Tong University Shanghai 200240 P. R. China

## Abstract

The optical performance of fused silica optics used in high-power lasers is known to depend not only on their surface damage resistance, but also on their surface quality. Previous studies have shown that good fused silica damage performance and surface quality can be achieved by the use of reactive ion etching (RIE), followed by HF-based wet shallow etching (3 μm). In this study, two kinds of HF-based etchants (aqueous HF and HF/NH_4_F solutions) were employed to investigate the effect of HF-based etching on the optical performance of reactive-ion-etched fused silica surfaces at various HF-based shallow etching depths. The results showed that the addition of NH_4_F to HF solution makes it possible to produce a high-quality optical surface with a high laser-induced damage threshold, which is strongly associated with the surface roughness and fluorescence defect density. Additionally, changing the HF-based etching depth over the range from 1 μm to 3 μm can affect the surface damage resistance and absorption performance of RIE-treated fused silica. The light-scattering results indicate that the point defect density plays an important role in the determination of the HF-based etching depth. Understanding these trends can enable the advantages of the combined technique of RIE and HF-based etching during the fabrication of high-quality fused silica optics.

## Abstract 2

The addition of NH_4_F to HF solution is important for producing a smooth fused silica surface with good laser damage resistance.

## Graphical abstract

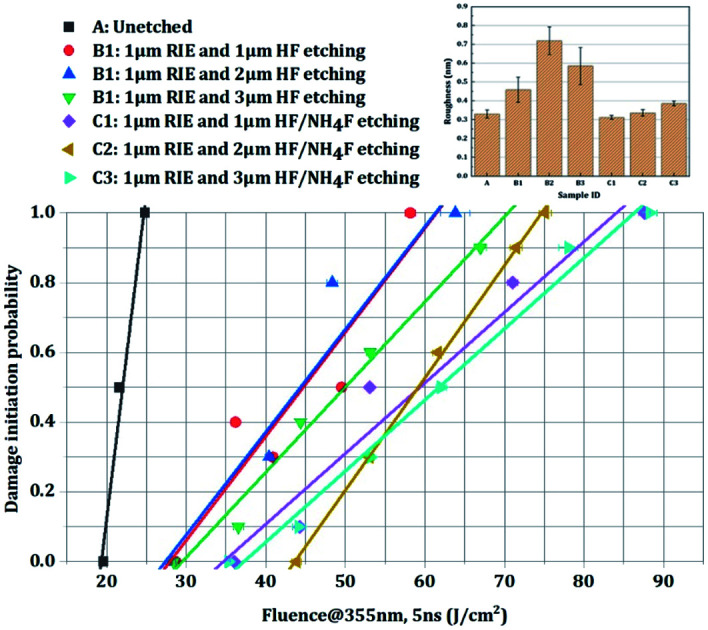

## Introduction

In high-power fusion-class laser facilities, the service life of fused silica optics is known to be severely limited by their surfaces, as laser-induced damage (LID) initiation and damage growth lead to unacceptable obscuration or even catastrophic failure of the optics.^1,2^ The LID of the fused silica surface, which typically scales with the laser fluence, results from the presence of extrinsic damage precursors located on the surface or within the subsurface layer of the optics.^3^ Photoactive impurities (*e.g.*, ceria) in the polishing redeposition layer^4,5^ as well as electric defects associated with a fractured surface,^3,6,7^ which are capable of strongly absorbing sub-band-gap light and spawning optical damage, have been identified as main laser-damage precursors. From a chemical-structure point of view, researchers have also reported that luminescent defects such as oxygen-deficient centers (ODCs) may possibly be associated with the first step of laser damage initiation.^8,9^ Additionally, recent studies have indicated that organic and inorganic residues at the nanoscale on the fused silica surface might be potential damage precursors at high fluence (*e.g.*, UV laser fluences >10 J cm^−2^ for 3 ns pulse duration).^10,11^

A possible approach to increase the laser-induced damage threshold (LIDT) of fused silica is to continually reduce the fractured subsurface defects such as scratches and microfractures, either through fine polishing or improved handling procedures.^7^ However, in practical terms, the progress in eliminating fractures is extremely limited. Another path to further reducing the number of damage precursors on a fused silica surface is the development of a whole-optic HF or HF/NH_4_F chemical etching process (such as AMP described by LLNL^12^). With the inception of this concept, this idea proved to be key to access a higher level of laser-induced damage resistance in fused silica. However, etching-related precursors might reappear when the material removal amount increases, which again reduces the LIDT.^13^ More importantly, this approach trades enhanced damage resistance for reduced surface quality properties, such as roughness and flatness due to HF-acid isotropically exposing scratches and leaving etching traces.^14–16^

Reactive ion etching (RIE), as developed in the semiconductor industry, is a prominent surface postprocessing technique that exploits the efficient removal of the material (such as silicon, silica, or metals) to produce a high-quality interface or surface profile at even the nanoscale.^17^ This approach has also prompted seminal demonstrations of the anisotropic elimination of the fractured defects in the subsurface layer of fused silica.^18,19^ However, the technological exploitation of RIE for the damage resistance enhancement of fused silica optics has proven to be impractical. This is because ion bombardment, radiation-induced bonding changes, and charge buildup can readily occur on the fused silica surface during the plasma etching process, forming chemical-structure defects and thus reducing the LIDT of the optics.^20,21^ Recently, we experimentally demonstrated that a RIE-treated defect layer can be efficiently removed by HF/NH_4_F wet shallow etching (3 μm removal amount). Moreover, this combined method proved to be technologically capable of both increasing the LIDT and smoothing the optical surface of fused silica.^22^ However, some key issues that may influence the effectiveness of this method remain unclear, such as the role of the added NH_4_F in HF acid solution during the combined treatment and the differences in the optical performance as a function of the HF and HF/NH_4_F shallow-etching depths.

In the following study, we investigate the effect of HF-based wet shallow etching on the optical performance of RIE-treated fused silica optics. The investigation includes a detailed comparison between two more-conventional etchants, aqueous HF and HF/NH_4_F. The laser-induced damage probability of the samples was obtained using a small-area laser testing system. Their surface morphology, roughness, optical transmission, fluorescence spectra, light-scattering imaging, and weak absorption were systematically characterized and analyzed. The results show that the addition of NH_4_F to HF solution is highly important for improving laser damage resistance, as well as surface quality, optical transmission, and FL characteristics. The mechanisms of the optical performance enhancement of the combined etched samples were subsequently revealed. These results may have important implications for control and mitigation of the laser-induced damage precursors on fused silica surfaces during the combined process of RIE and HF-based wet etching.

## Experimental

### Sample preparation

Seven square polished fused silica samples (Corning 7980) were utilized for the etching experiments. All the samples (named A, B1, B2, B3, C1, C2, and C3) were typically 50 mm in side-length and 5 mm in thickness. The polishing of each sample was conducted *via* the same process utilizing 0.4 μm CeO_2_ abrasives. Sample A was not etched to be kept as a reference. For other samples, two types of wet etching processes were performed using HF or HF/NH_4_F chemical solution.

### Etching experiments

In this study, an optimized shallow RIE (1 μm removal amount) pretreatment was performed on the fused silica sample surfaces in a parallel-plate discharge plasma etcher. A gas mixture consisting of ultra-pure CHF_3_ and Ar in a fixed ratio was ionized using the optimized plasma etching process. In this process, fluorine-containing neutral species (such as CF_3_ and CF_2_) and reactive radicals (such as F^−^) will be formed to react with the silica, and Ar plasma can stabilize the electron energy distribution. For each sample, only one side (as an exit surface for the damage testing) was treated using the RIE process. The etching rate was fixed to 35 nm min^−1^. The experimental setup for RIE can be found elsewhere.^23^

**Fig. 1:**
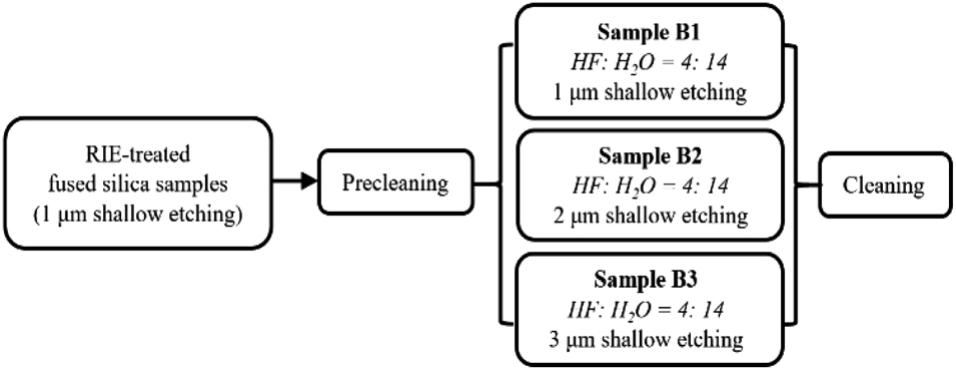
Preparation of the samples for HF shallow etching with different depths.

**Fig. 2:**
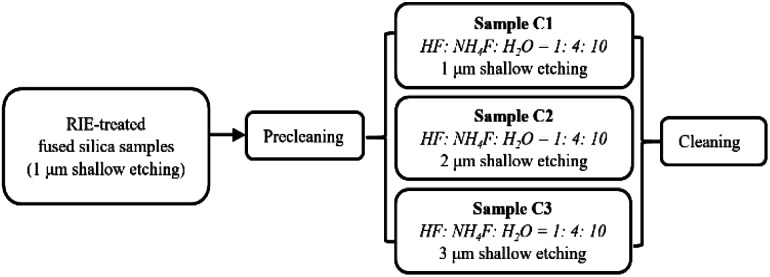
Preparation of the samples for HF/NH_4_F shallow etching with different depths.

The RIE-pretreated samples were then treated again using dynamic cleaning and an HF-based chemical etching technique (described as DCE in our previous study^22^). Two sets of three samples were treated with different HF-based etching depths (1 μm, 2 μm, and 3 μm, respectively), as summarized in Fig. 1 and 2. The first set (samples B1, B2, and B3) are the polished surfaces treated with RIE and HF etching, while the second set (samples C1, C2, and C3) are the polished surfaces that underwent RIE followed by HF/NH_4_F etching. The HF/NH_4_F etchant, which was more commonly used in our previous study, consists of HF (49 wt%), NH_4_F (30 wt%), and H_2_O (ultra-pure water) with a volume ratio of 1 : 4 : 10. We chose an HF-only (49 wt%) solution containing approximately 78 vol% H_2_O because its etching rate (100 nm min^−1^) was equal to that of the HF/NH_4_F solution we typically used.

### Damage tests and characterization

The damage tests of the fused silica samples prepared using our combined RIE and DCE processes were performed using a Q-switched Nd:YAG laser operating at 355 nm. Unlike the damage test system used in our previous study,^23^ this system uses a relatively small-area laser beam focused at the exit surface (around 0.25 mm in diameter) to ensure that all the etched surfaces can be laser-damaged within the available laser fluence range. The temporal profile of the laser beam was near-Gaussian with a full width at half max pulse duration of approximately 5 ns. The output fluence of the laser pulse was adjustable as needed for the execution of the experiments. The tests were performed using 1-on-1 methodology following ISO 21254.^24^ Twenty irradiation sites were randomly chosen for each laser fluence. The damage locations were observed using an automated microscope that could provide individual damage site images with a resolution of 0.2 μm.

We used optical microscopy (OLYMPUS MX61, Japan) to observe the morphologies of the etched surfaces. The morphologies of the laser-damaged samples were also observed. The magnification during the observation was controlled to be 500×. The surface roughness *R*_q_ (RMS, root mean square) value of each fused silica sample was measured using a confocal white-light interferometer (SENSOFAR PLU NEOX 3D, Spain) with a 768 pixel × 576 pixel resolution. A total of five measurements with an area of 250 μm × 180 μm were randomly chosen to obtain the mean roughness value of each sample.

Various scattering (SC) characteristics of the combined etched fused silica surfaces were measured using a self-developed laser-induced fluorescence microscopy system. The system allows simultaneous measurement of SC features. The SC signals on the sample surfaces were excited by a green laser with a wavelength of 532 nm. We obtained a large 3 mm × 3 mm image by stitching together 25 (5 × 5) sub-images. To obtain statistical results, the total size, standard deviation (std. dev.) of the sizes, and the total number of SC objects were determined using the analysis software Image-Pro.

The transmission of the samples treated with different combined etching processes was measured using a UV/VIS-spectrometer (Lambda 950 from PerkinElmer, Inc.) to investigate the effect of the HF-based etching process on the optical absorption of the samples. The spectrometer has a detection wavelength range of 200 nm to 800 nm. Five regions were randomly chosen on each sample surface to obtain a convincing result. We fixed the samples on a stage in the same position and angle to ensure that all the transmission results were comparable.

Fluorescence (FL) emission spectra can be used to determine the typical chemical structure defects (*e.g.*, ODCs) on fused silica surfaces. In this study, we measured the FL emission spectra of the etched samples using a xenon lamp as the excitation source with a wavelength of 210 nm (∼5.9 eV). The exciting laser beam was focused on the sample surfaces with a 45° incidence angle. The FL emission signal was detected through a photomultiplier. The position and angle of each sample were fixed to be consistent. A detailed description of the FL spectra measurement has been reported elsewhere.^25^

## Results

**Fig. 3:**
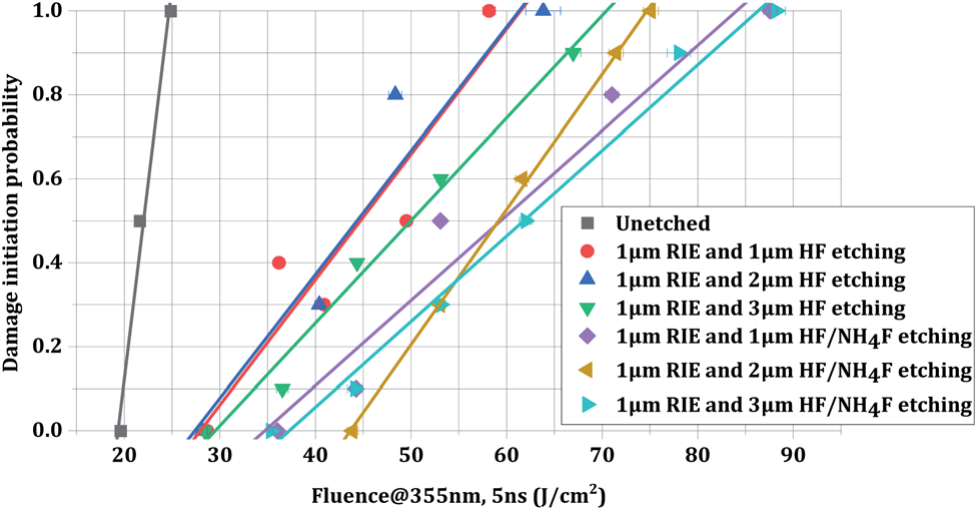
Laser damage probability of various surfaces treated with different HF-based etching processes. The quality factor (peak energy/average energy) of the laser beam spot was approximately 2.2. The energy density uncertainty of the testing system is 4.5%.

After RIE and HF-based etching, the treated samples and the as-polished sample were subjected to the LIDT test. Fig. 3 shows the relationship between the laser fluence and the 1-on-1 damage probability of the samples. Similarly to in our previous work,^23,25^ the damage threshold of the combined etched surface was much higher than that of the unetched surface. This great improvement was attributed to the effective removal of the subsurface damage layer, which contains abrasive residues and other polishing-induced contaminants. It can also be noted from the figure that the second set of samples (those treated with RIE followed by HF/NH_4_F etching) exhibited an increased LIDT compared to the first set of samples (those treated with RIE followed by HF etching). The results indicated that the addition of NH_4_F to the HF solution was beneficial for improving the LIDT of fused silica.

Table 1 shows a summary of the damage probabilities of the two sets of combined etched samples, as well as that of the unetched sample. From the data in this table, more interesting information can be obtained. The surfaces treated with RIE followed by HF etching at three different etching depths showed little difference among their 0% probability LIDT (27.8 J cm^−2^, 27.4 J cm^−2^, and 29.5 J cm^−2^, respectively) and 100% probability LIDT (61.5 J cm^−2^, 61.3 J cm^−2^, and 70.6 J cm^−2^). For the surfaces treated with RIE followed by HF/NH_4_F etching, the 0% probability damage threshold first increased and then decreased with increasing etching depth. The greatest improvement in the 0% probability LIDT was obtained at a 2 μm HF/NH_4_F etching depth (43.7 J cm^−2^), while that in the 100% probability LIDT was obtained after the sample surface underwent 3 μm HF/NH_4_F etching (86.5 J cm^−2^).

**Table 1:** Damage probability of each fused silica sample

Sample	0% probability damage threshold (J cm^−2^)	100% probability damage threshold (J cm^−2^)
A (unetched)	19.2	24.6
B1 (1 μm RIE and 1 μm HF etching)	27.8	61.5
B2 (1 μm RIE and 2 μm HF etching)	27.4	61.3
B3 (1 μm RIE and 3 μm HF etching)	29.5	70.6
C1 (1 μm RIE and 1 μm HF/NH_4_F etching)	34.8	83.9
C2 (1 μm RIE and 2 μm HF/NH_4_F etching)	43.7	74.6
C3 (1 μm RIE and 3 μm HF/NH_4_F etching)	37.3	86.5

**Fig. 4:**
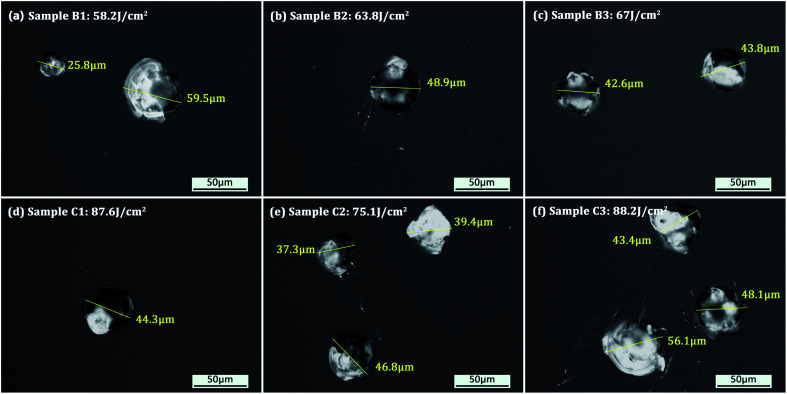
Morphology of 100%-probability laser-damaged locations of the two sets of the combined etched samples. The first row of images (a–c) shows the damage sites on the HF-etched fused silica surfaces. The second row (d–f) shows the damage sites on the HF/NH_4_F-etched fused silica surfaces. The laser fluence for each sample is noted. The yellow line across each damage crater indicates the approximate size (ranging from 25.8 μm to 59.5 μm) of the crater.

The surface morphologies of the 100%-probability laser-damaged locations for the two sets of the combined etched samples are illustrated in Fig. 4. No obvious differences in the damage morphologies of the samples were observed, regardless of which HF-based etchant or etching depth was chosen. The morphologies of the damage sites are generally smooth and disperse, and exhibit small microscale ablation craters (∼20 μm to ∼60 μm wide), suggesting that the damage precursors were relatively homogeneous. The formation of these smooth craters might be attributed to the energy deposition and shockwave acceleration at high laser fluence.^26^ In this case, the molten residues were substantially removed from the damaged craters. Additionally, slight amounts of ejected materials were observed near the edges of several craters (see Fig. 4(b), (e), and (f)), which was probably due to redeposition after fast ablation and vaporization of the materials. In Fig. 4(f), it can also be noticed that the damage produced plastic deformation near the craters.

**Fig. 5:**
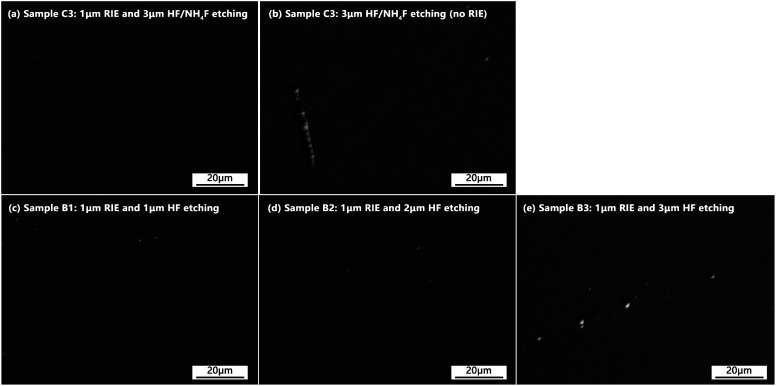
Optical micrographs showing representative surface morphologies of the etched samples. (a) Sample C3 (exit surface): 1 μm RIE and 3 μm HF/NH_4_F etching, (b) sample C3 (entrance surface): 3 μm HF/NH_4_F etching, (c) sample B1: 1 μm RIE and 1 μm HF etching, (d) sample B2: 1 μm RIE and 2 μm HF etching, and (e) sample B3: 1 μm RIE and 3 μm HF etching.

Fig. 5 shows representative surface morphologies (undamaged locations) of the etched fused silica samples (samples C3, B1, B2, and B3). For sample C3, the morphologies of both the laser entrance (3 μm HF/NH_4_F etching) and exit surfaces (combined etching) are given. The morphology results of samples C1 and C2 are not given because there were no obvious differences among the three HF/NH_4_F-etched sample surfaces (at least for the observed results using 500× optical microscopy, see ESI†). It can be seen from the figure that the morphologies of the surfaces treated with the combined processes are very similar to each other. All the treated surfaces were very smooth without visible scratches or micro-cracks except for several low-density micro-pits. On the contrary, the entrance surface treated with only 3 μm HF/NH_4_F etching presents a large number of scratches, which had been passivated by the HF/NH_4_F solution, as shown in Fig. 5(b). The results suggested that the shallow combined etching process was very effective for tracelessly removing the fractured defects in the subsurface layer of the polished fused silica optics. However, the etched surfaces treated with RIE followed by HF etching exhibited slightly more micro-pits, which was indicative of the fact that this HF etching step might treat the subsurface fractured defects more isotropically. It is difficult to obtain the distribution of these isolated point defects; however, the SC imaging shown below can be used as an efficient tool for examining them.

**Fig. 6:**
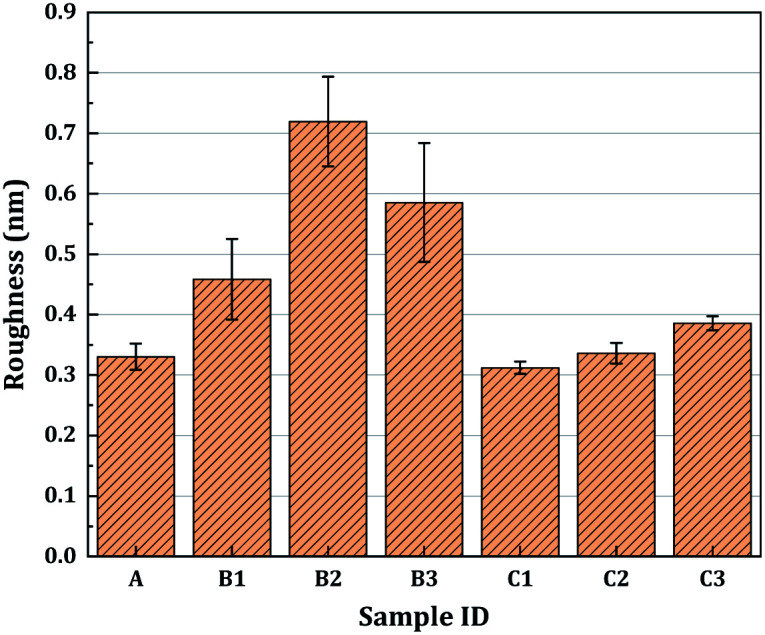
Roughness *R*_q_ (at the sub-millimeter scale) of the as-polished surface and surfaces treated with RIE followed by HF or HF/NH_4_F wet etching with different etching depths. The error bars indicate the standard deviation of the measured *R*_q_ values.

We then compared the surface roughness of the two sets of treated fused silica samples after RIE followed by HF-based etching, as well as that of the as-polished sample. In Fig. 6, each bar represents the mean *R*_q_ value of all the measured points on each sample surface. The unetched sample surface had a relatively low roughness (0.33 nm). The surface roughnesses of the samples treated with RIE followed by HF etching increased significantly (from 0.46 nm to 0.72 nm) as the etching depth was increased to 2 μm, but decreased to 0.59 nm after 3 μm etching. For the HF/NH_4_F etched surfaces, the surface roughness increased slightly with the etching depth, and the variations in the *R*_q_ value were less than 0.1 nm. The maximum *R*_q_ value was only 0.39 nm (sample C3).

**Fig. 7:**
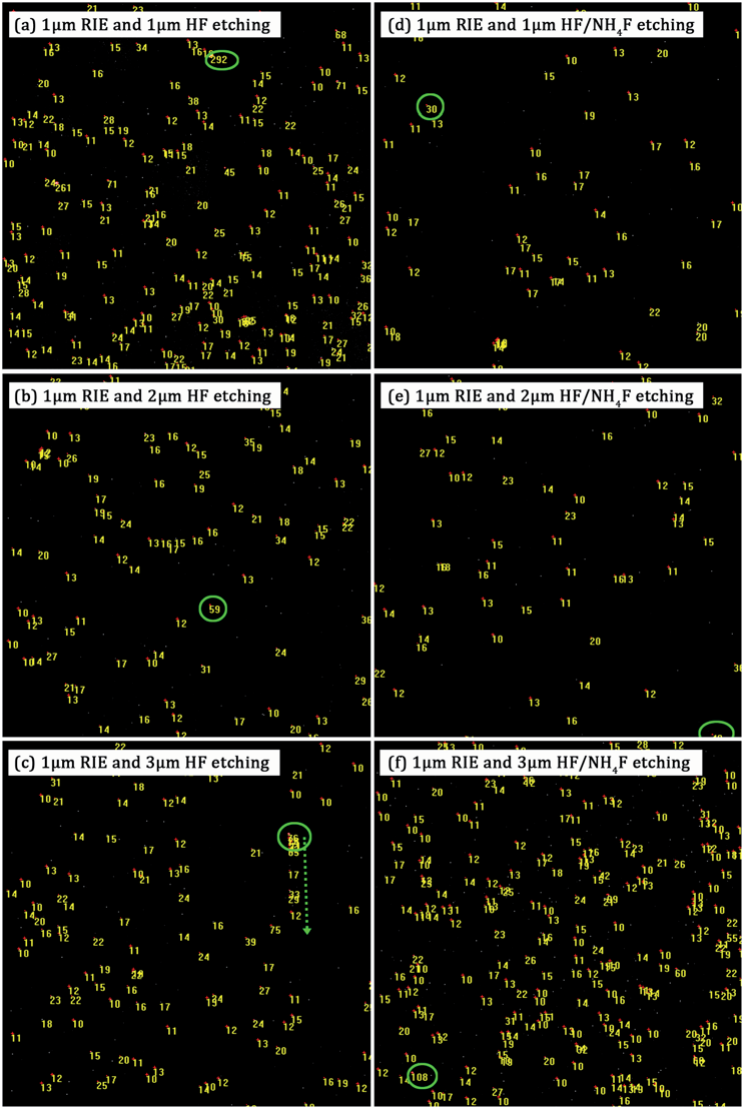
SC images of the samples treated with RIE followed by HF-based etching. Left column (a–c): RIE and HF etching with removal amounts of 1 μm, 2 μm, and 3 μm. Right column (d–f): RIE and HF/NH_4_F etching with removal amounts of 1 μm, 2 μm, and 3 μm. Each image measures 3 mm per side. Yellow numbers on each image indicate the sizes (number of pixels) of the SC objects. Only objects with a size larger than 10 pixels were marked. The largest-sized SC object on each sample surface is also circled in green.

High-resolution SC imaging was used to observe microscopic features on the HF-based etched fused silica surfaces that were pretreated using RIE, as shown in Fig. 7. We used the software Image-Pro to obtain the size of each SC object by calculating the pixels it comprised. The left column represents the samples treated with the RIE and HF etching process, while the right column represents the samples treated with the RIE and HF/NH_4_F etching process. It was noted that many point features were visible on all the observed sample surfaces, regardless of which HF-based etching process or removal amount was chosen. The point defects were probably due to the presence of embedded particles in the subsurface layer and even the silica matrix. Because HF acid solution has a higher etching rate toward these particles than toward the silica matrix, the particles were first detached from the surface, leaving typical traces of pits. An interesting phenomenon observed from the SC images was that the densities of the point defects on the different treated sample surfaces were different. It was noted that, for the left column, the sample surfaces treated with RIE followed by 1 μm HF etching had the highest density of point defects. On the contrary, for the right column, the density of point defects on the surface treated with RIE followed by 3 μm HF/NH_4_F etching was the highest. Except for samples B1 and C3, all others had a very similar density of point defects.

**Fig. 8:**
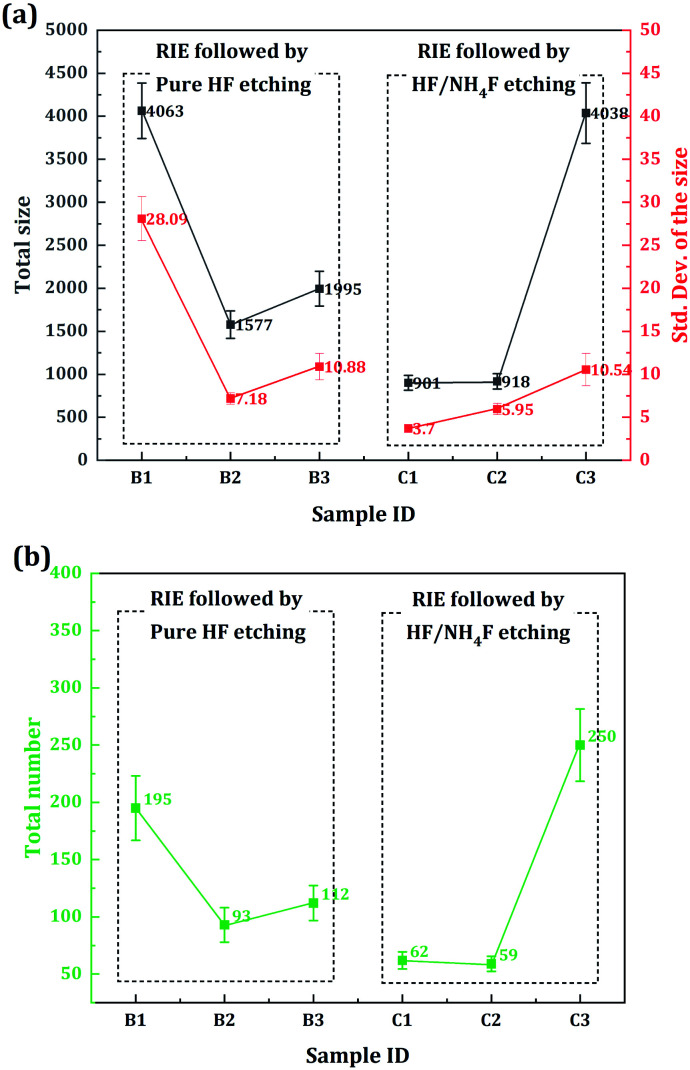
Characteristics of the point defects on the etched sample surfaces. The gray, red, and green squares indicate the total size, standard deviation (std. dev.) of the size, and the total number of SC objects, respectively. The raw data and corresponding statistical data of the SC images can be seen in the ESI.†

To determine the characteristics of the point defects on the sample surfaces, we further extracted the total size, the standard deviation (std. dev.) of the size, and the total number of SC objects. As shown in Fig. 8, all the parameters presented similar trends for each of the HF-based etching processes. Additional information can be obtained through comparative analysis. First, samples B1 and C3 had the largest total sizes (nearly the same, ∼4100 pixels) of point defects. Second, for the same etching depth, the std. dev. of the sample surface treated with RIE followed by HF/NH_4_F etching was lower than that of the surface treated with RIE followed by HF etching. Third, the total number of point defects on sample B1 was a little lower than that on sample C3. However, the other HF/NH_4_F-etched samples (samples C1 and C2) had a relatively low total number of point defects compared to the other HF-etched samples (samples B2 and B3).

**Fig. 9:**
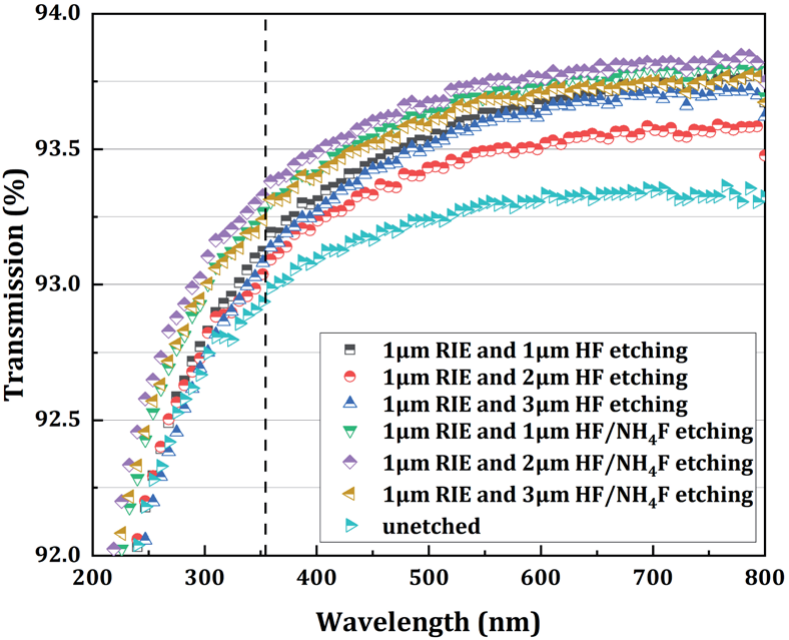
Spectral transmission of the fused silica samples treated with RIE followed by HF-based etching. The spectral transmission of the unetched sample is used as a reference.

Fig. 9 shows the spectral transmission of the fused silica samples treated with RIE followed by HF-based etching. It was noted that all the combined-etched samples had a relatively high optical transmission compared to the unetched sample. It was also noted that the addition of NH_4_F to the HF solution led to a slight increase in transmission, corresponding to a decrease in the absorption and scattering of the incident laser on the treated fused silica samples. This trend can be observed for all the samples treated with RIE followed by HF/NH_4_F etching, but was most significant in the case of the 2 μm HF/NH_4_F etching depth (sample C2). In the UV wavelength range (*e.g.*, from the lowest measured wavelength, 190 nm, up to approximately 400 nm), this observed increase in transmission was more obvious. Additionally, it can also be noted that the transmission of the sample treated with RIE followed by 2 μm HF etching (sample B2) decreased strongly, especially in the visible range. The decrease in the transmission might be due to the plasma-induced and chemical-etching-driven formation of chemical structure defects,^27^ such as the ODCs mentioned in the Introduction.

Finally, we performed FL spectra analysis for all the combined-etched samples as well as the unetched sample. It was noted that all the FL spectra had a very similar trend with the excitation wavelength. As expected, the unetched sample had the highest observed FL peak intensity. It can be also noted that the addition of NH_4_F to the HF solution did not appear to cause a change in the FL defect type. Three main FL bands can be resolved from the measured emission spectra in the range of 300–800 nm. The 3.5 eV band is well known, and is attributed to ODC defects, while the 1.89 eV band is attributed to NBOHC defects. The other FL band centered at 2.93 eV is likely O^2−^. Compared to the first set of samples (samples B1, B2, and B3), the second set of samples (samples C1, C2, and C3) had a much lower fluorescence intensity. For the first set of samples, the HF etching depth had a weak effect on the peak intensity of all the FL bands. For the second set of samples, however, we found that the FL peak intensity was highly dependent on the HF/NH_4_F etching depth. The intensities of the ODC and NBOHC defect peaks decreased with increasing HF/NH_4_F etching depth. The results indicated that HF/NH_4_F etching at a greater depth could cause a lower concentration of FL defects in the fused silica network.

## Discussion

The combined treatment with RIE and HF-based wet etching is an attractive method to effectively remove the surface damage precursors of fused silica optics. As in the conventional aqueous HF or HF/NH_4_F etching processes, preventing the redeposition of the reaction products (SiF_6_^2−^) on the etched surface of the fused silica is still a key challenge facing this combined process. In addition to using multi-frequency agitation and spray rinsing to increase the mass transport of the reaction products away from the etched surface, controlling the chemistry and decreasing the HF-based etching depth are also important approaches to minimize hexafluorosilicate redeposition, as well as to constrain the quality degradation of the optical surface. This work involved two main investigations to help in understanding how the HF-based wet shallow etching influences the optical performance of the RIE-treated fused silica surface. First, we compared HF etching with HF/NH_4_F etching to investigate the effect of the last etching step on the laser-induced damage performance and surface quality of the fused silica. Secondly, we investigated the evolution of the defects on the combined-etched fused silica surfaces by changing the HF-based shallow etching depth (1 μm, 2 μm, and 3 μm).

As shown in Fig. 3 and Table 1, a significant improvement in the laser damage threshold was observed for all the samples treated with the combined etching process. The morphology of the damage sites shown in Fig. 4 also indicated that the combined etching can form a relatively pure fused silica optical surface with a homogeneous damage precursor type. This improvement has been largely attributed to the traceless removal of the fractured subsurface defects, which either absorb laser energy or cause field intensification.^22^ The etchants utilized in the wet etching play an important role in fully exploiting this technique. Combined treatment using 1 μm RIE and shallow HF etching can achieve at most a 1.60-fold enhancement in the 0% probability damage threshold (sample B3). A further increase in damage threshold (up to 2.3 times for sample C2) can be created by adding a certain amount of NH_4_F to the HF solution. Based on the previous study by Suratwala,^13^ ammonium hexafluorosilicate [(NH_4_)_2_SiF_6_] precipitate can form when using HF/NH_4_F as an etchant, and the solubility of SiF_6_^2−^ scales inversely with the square of the ammonium fluoride concentration. The precipitation reaction and corresponding solubility of SiF_6_^2−^ are given by

1 SiF_6_^2−^(aq) + 2NH_4_^+^(aq) → (NH_4_)_2_SiF_6_(solid),

and

**Fig. 10:**
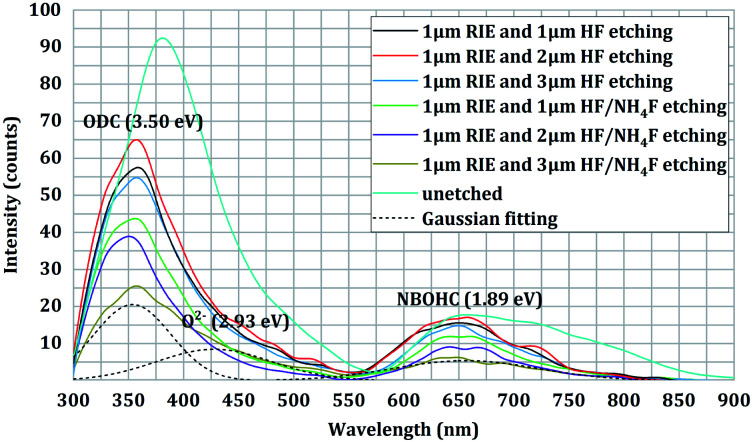
Comparison of the FL spectra under 210 nm (5.9 eV) excitation for the combined-etched samples. The black dotted lines present the three Gaussian components of the spectrum deconvolution for the sample surfaces treated with 1 μm RIE and 3 μm HF/NH_4_F etching. The FL spectrum of the unetched sample is used as a reference.

2 *S*_SiF_6_^2−^_ = 1.94 (mol per liter)^3^/[NH_4_F]^2^,

where *S*_SiF_6_^2−^_ is the solubility of SiF_6_^2−^ and the NH_4_F is assumed to completely dissociate in the HF/NH_4_F solution. Therefore, the need for NH_4_F during the HF etching to achieve a high laser damage threshold initially surprised us. Several mechanisms have been proposed to explain the need for adding NH_4_F to the HF solution: (1) HF/NH_4_F can stabilize the etching rate and offer comparatively lower vapor pressures of HF, which further influences the surface quality of the optics. As shown in Fig. 6, the surface roughness of the samples etched in HF solution is substantially higher than for those in HF/NH_4_F solution. The rough surface has a high propensity to house trace redeposition due to surface-tension-induced fluid pinning, especially during the drying process.^13^ Additionally, the local surface absorption and light-scattering could also be enhanced, since the spectral transmission of the HF-etched samples decreased obviously (see Fig. 9). All these factors would lead to an increase in LID probability. Thus, the decrease in the surface roughness resulting from the addition of the NH_4_F might be a key factor in enhancing the laser damage resistance of the etched fused silica. (2) The activation energy for the dissolution of fused silica in the HF/NH_4_F solution is significantly higher than that in the HF solution.^28^ A more stable SiO_2_ network might exist after the HF/NH_4_F etching. The FL spectra results give a clear insight into the way in which NH_4_F affects the intensity of chemical structure defects such as ODC, O^2−^, and NBOHC during the HF-based wet etching of fused silica (see Fig. 10). The concentration of the chemical structure defects is shown to be one of the important factors influencing the creation of high LIDT surfaces of fused silica.

From an etching depth point of view, no obvious difference in 0% probability damage threshold can be observed for the samples treated with RIE followed by HF etching. However, there is an association between the etching depth and the LIDT of the samples treated with RIE followed by HF/NH_4_F etching (see Fig. 3 and Table 1). It is well understood that the 0% probability damage threshold increases when the HF/NH_4_F etching depth is increased from 1 μm to 2 μm, but its subsequent decrease at 3 μm etching depth confused us, especially as a significant decrease in the FL defect intensity of the sample C3 is observed (again, see Fig. 10). The SC imaging results provide important clues to the determination of the defects, as well as the corresponding mechanism. As shown in Fig. 7 and 8, high-density point defects exist on the 3 μm HF/NH_4_F-etched fused silica surface. The total size and number of the point defects on sample C3 are approximately 4.4 times and 4.2 times higher than that for sample C2. These observed point defects may be attributed to isolated round pits more randomly distributed on the sample surface, which are typically several or tens of micrometers in diameter. As discussed above, high-density physical structure defects can act as traps for the redeposition of the reaction products during the etching and drying processes. To maximize the surface damage resistance of fused silica, the density of the point defects that can give rise to the SC effect should be minimized by precisely controlling the HF/NH_4_F etching depth. It is considerably difficult to observe the majority of the point defects using an optical microscope (see Fig. 5), at least at the present resolution of 500×. Hence, the SC imaging technique can offer a powerful tool for optimizing the etching depth during the combined RIE and DCE treatment.

**Fig. 11:**
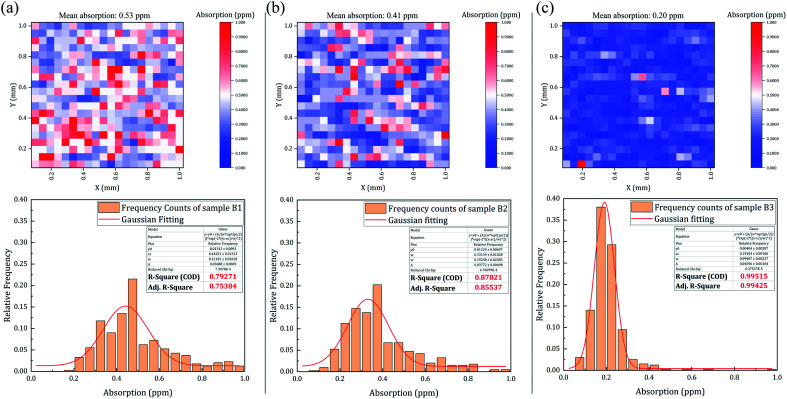
Surface weak absorption mapping and corresponding statistical data (relative frequency) of the samples treated with RIE followed by HF etching (samples B1, B2, and B3). The measurements were conducted on 10 mm × 10 mm scanning regions with a 0.05 mm step size for 400 testing points.

Although the HF etching depth had a weak effect on the damage probability in the present study, the distribution of the defects and the corresponding absorption on the sample surface might change with the etching depth. For the three samples treated with RIE followed by HF etching, it can be found from Fig. 3 that sample B3 had the best linear fitting relationship compared to the other two samples (see the fitting line and corresponding data points for each sample). A similar effect can be also observed on the surfaces treated with RIE followed by NH_4_F/HF etching. To further understand the effect of the HF etching depth on the optical performance of the combined-etched fused silica samples, we measured the weak absorption of the optical surfaces treated with RIE followed by HF etching (samples B1, B2, and B3), as shown in Fig. 11. The measurement was conducted using our self-developed photo-thermal deflection system based on a photo-thermal common-path interferometer. The pump beam was a 355 nm quasi-continuous laser with an output power of ∼1.1 W. The beam spot was focused on the sample surface with a diameter (1/*e*^2^) of ∼20 μm. The probe beam was a modulated He–Ne laser that overlapped with the pump laser on the tested sample surface. A detailed description of the system can be found elsewhere.^29^ 400 sites in a 10 mm × 10 mm region on each sample surface were chosen for the measurement. It can be noted that the mean absorption of the whole region trended to be a lower level when increasing the HF-etching depth. The relative frequencies of the absorption values were then analyzed and Gaussian-fitted. It was noted that the fitting relationship between the two variables became stronger as the HF-etching depth was increased (see the *R*-square coefficients). The results indicated that deep HF etching would make the fused silica surface more stable and uniform.

**Fig. 12:**
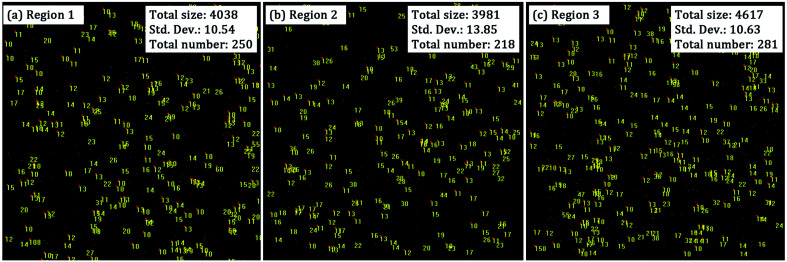
SC images of three different regions of the sample treated with 1 μm RIE followed by 3 μm HF etching (sample C3). Yellow numbers on each image indicate the sizes (number of pixels) of the SC objects. Only objects with a size larger than 10 pixels were marked. The total size, the std. dev. of the sizes, and the total number of the SC objects for each region are also noted. Also, the raw data and the corresponding statistical data can be seen in the ESI.†

One issue not yet addressed is whether the difference in the point defect density is due to the inhomogeneity of the material. We thus chose another two regions on the surface of sample C3 to compare the SC results (regions 2 and 3) with those we had obtained before (region 1). As shown in Fig. 12, similar SC features were observed for the three regions on the same sample surface, suggesting that the material on the fused silica surface was relatively homogeneous. We would not associate this variation with the HF-based etching depth, because the surface treated with 1 μm RIE followed by 1 μm HF etching (sample B1) also had a high point defect density. More evidence, such as the SC results of a series of samples treated with the same combined etching process, will be obtained in future work.

## Conclusions

The combined processes of RIE and aqueous HF-based etching have been shown to significantly improve the laser damage threshold of polished fused silica optics. The objective of this work was to advance the understanding of the influence of HF-based shallow (≤3 μm) wet etching on the optical performance of RIE-treated fused silica optical surfaces and to ensure optics suitable for practical applications in high-power-peak laser systems. Two kinds of etchants, HF and HF/NH_4_F, which exhibit different features, were investigated as case studies. The results revealed that the addition of NH_4_F to HF solution is conducive to improving not only the laser damage resistance, but also other performance parameters such as surface quality, optical transmission, and FL characteristics. The surface roughness and FL defect intensity play important roles in the LIDT enhancement of the combined-etched samples. An optimal initial LIDT level was observed when the HF/NH_4_F etching depth was matched with the 1 μm RIE depth, which was strongly associated with the point defects observed by light-scattering imaging.

These results may have important implications for the control and mitigation of the laser-induced damage precursors on the surface of fused silica. Future work will seek to clarify the possible origin of the point defects featuring SC and further reveal the nature of the effect of their density on the laser damage behavior in achieving high-quality fused silica optics by using the combined etching process.

## Author contributions

Laixi Sun: conceptualization, methodology, software, validation, formal analysis, investigation, writing – original draft, writing – review & editing. Ting Shao: resources, data curation, writing – review & editing. Xinda Zhou: resources, data curation. Weihua Li: resources, data curation. Fenfei Li: resources, data curation. Xin Ye: methodology, resources, data curation. Jin Huang: resources, data curation. Shufan Chen: resources, data curation. Bo Li: resources, data curation. Liming Yang: conceptualization, methodology, formal analysis, resources, data curation, writing – review & editing. Wanguo Zheng: conceptualization, methodology, formal analysis, resources, data curation, writing – review & editing.

## Conflicts of interest

There are no conflicts to declare.

## Supplementary Material

RA-011-D1RA04174F-s001
