# Supplementary material for: Understanding the effect of HF-based wet shallow etching on optical performance of reactive-ion-etched fused silica optics
Source: RSC Adv. 2021 Sep 1;11(47):29323–32. doi: 10.1039/d1ra04174f (PMC9040614; doi:10.1039/d1ra04174f)

Electronic Supplementary Material (ESI) for RSC Advances.
This journal is © The Royal Society of Chemistry 2021

Figure S1. Surface morphologies of the samples treated with RIE followed by HF/NH_4F etching. Left: 1-μm RIE and 1-μm HF/NH_4F etching, middle: 1-μm RIE and 2-μm HF/NH_4F etching, right: 1-μm RIE and 3-μm HF/NH_4F etching. The results showed that there was no obvious difference in surface morphology among the three HF/NH_4F-etched sample surfaces.

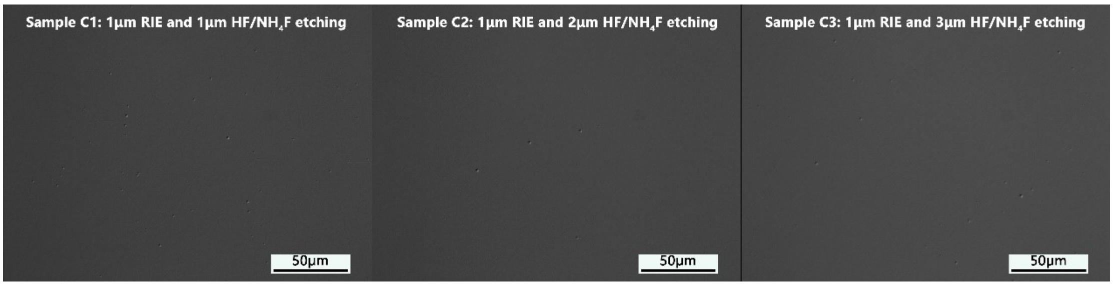

Figure S2. Raw data of the SC imaging for the six combined-etched samples. Left column: RIE and HF etching with removal amount of 1 μm, 2 μm, and 3 μm. Right column: RIE and HF/NH_4F etching with removal amounts of 1 μm, 2 μm, and 3 μm.

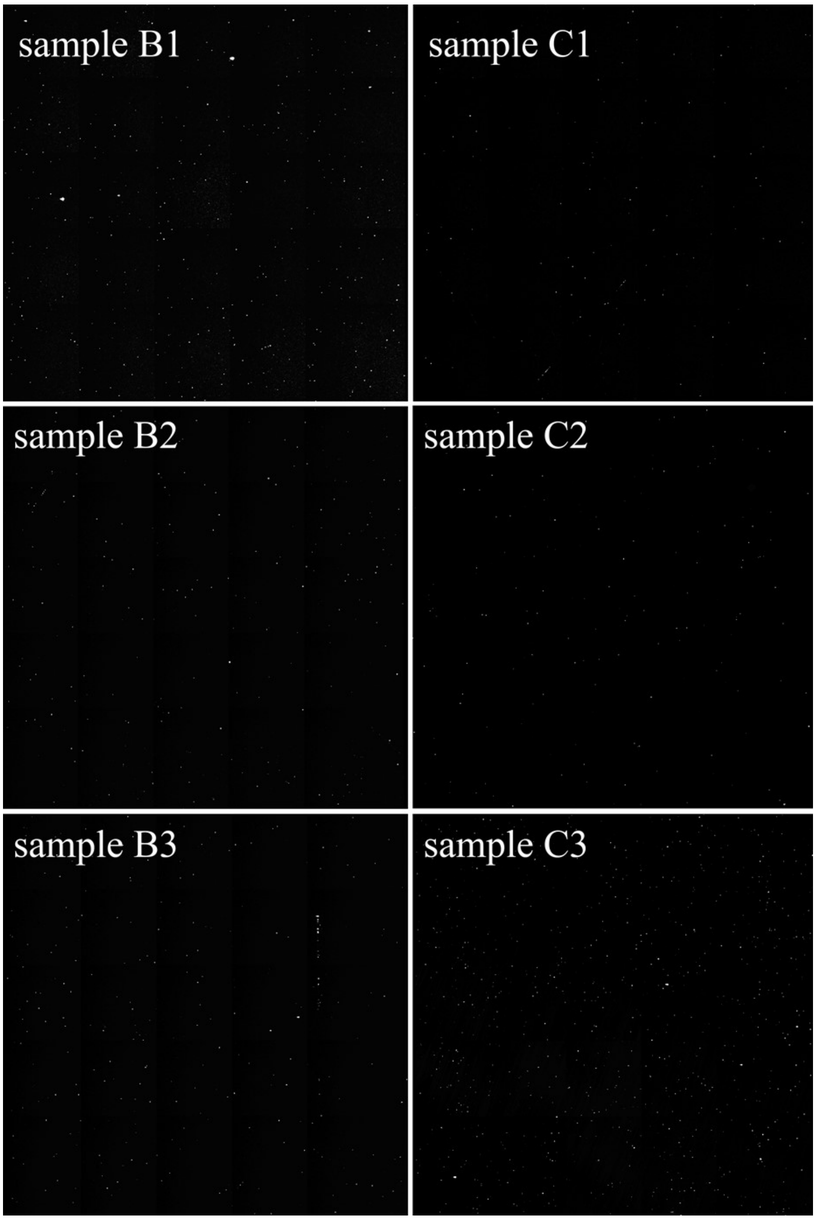

Figure S3. Statistical data of the SC imaging for the six combined-etched samples. Left column: RIE and HF etching with removal amount of 1 μm, 2 μm, and 3 μm. Right column: RIE and HF/NH_4F etching with removal amounts of 1 μm, 2 μm, and 3 μm.

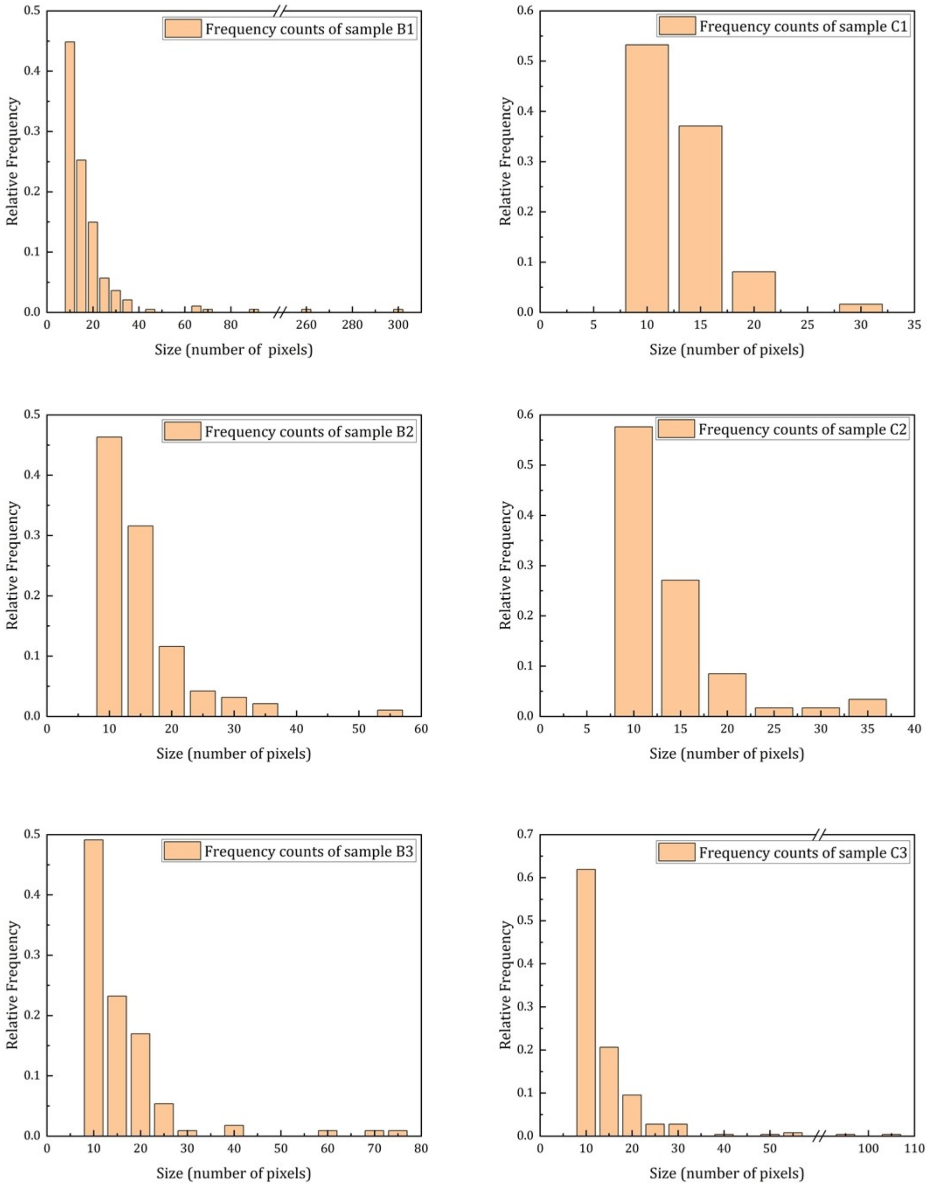

Figure S4. Raw data of the SC imaging for sample C3 (1-μm RIE and 3-μm HF/NH_4F etching).

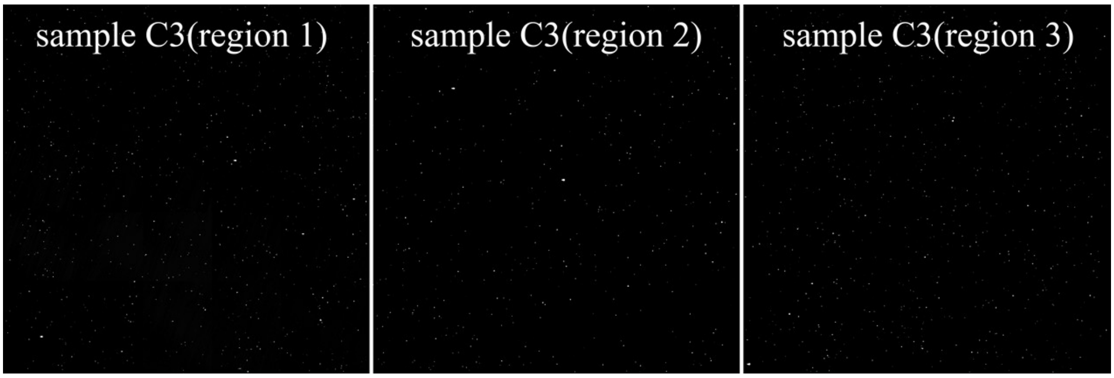

Figure S5. Statistical data of the SC imaging for sample C3 (1-μm RIE and 3-μm HF/NH_4F etching).

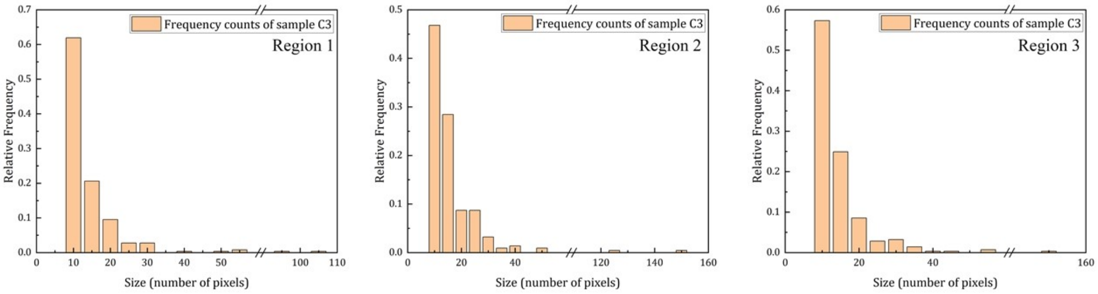

Supplement: RA-011-D1RA04174F-s001 [file RA-011-D1RA04174F-s001.pdf]
